# Rehabilitation Progress in Patients Following Surgery for Acute Stanford Type A Aortic Dissection Extending Beyond the Ascending Aorta

**DOI:** 10.3390/jcm14010197

**Published:** 2025-01-01

**Authors:** Joanna Nowak, Mariusz Listewnik, Aleksandra Rył, Jerzy Pacholewicz, Iwona Rotter

**Affiliations:** 1 Department of Cardiac Rehabilitation, Cardiac Surgery Clinic, University Hospital No. 2, 70-111 Szczecin, Poland; 2 Department of Medical Rehabilitation and Clinical Physiotherapy, Pomeranian Medical University, 70-204 Szczecin, Poland; aleksandra.ryl@pum.edu.pl (A.R.); iwona.rotter@pum.edu.pl (I.R.); 3 Cardiac Surgery Clinic, Pomeranian Medical University, 70-204 Szczecin, Polandjerzy.pacholewicz@pum.edu.pl (J.P.)

**Keywords:** acute aortic dissection, aortic aneurysm, cardiac rehabilitation, physical exercises

## Abstract

**Background:** The objective of this study was to assess the course of rehabilitation of patients hospitalized in the cardiac rehabilitation unit after surgery for acute Stanford type A aortic dissection, extending beyond the ascending aorta, and comparing these findings with those for patients who, after the same type of surgery, had no remaining dissection. The aim was to develop an optimal cardiac rehabilitation model for this patient population, given the lack of clear guidelines. Additionally, the study aimed to evaluate their one-year survival. **Methods:** The study included patients referred to the cardiac rehabilitation unit after surgery for acute Stanford type A aortic dissection extending beyond the ascending aorta (a total of 25 patients). The study group was compared with a cohort of patients (a total of 58) who underwent similar cardiac surgery. The main difference was the absence of residual aortic dissection. All patients were assessed on admission to the cardiac rehabilitation unit and at discharge using the 6-min walk test and the Tinetti test. All patients underwent 2–3 weeks of rehabilitation following the same protocol; however, the study group had significantly reduced exercise loads. **Results:** Patients in the study group were admitted to the cardiac rehabilitation unit in a significantly worse functional status compared to the comparison group, but the final assessment showed comparable results for the Tinetti test and the 6-min walk test. There were no differences in one-year survival between the two groups. **Conclusions:** Early postoperative cardiac rehabilitation in patients after acute aortic dissection significantly improves the functional status of this patient group, and is safe.

## 1. Introduction

Acute aortic dissection is characterized by the presence of an intimal flap separating the true from the false lumen, which may or may not communicate [1]. When the intimal layer tears and blood infiltrates the dissection space, the aorta may rupture if the adventitia also ruptures. Alternatively, blood from the dissection space may re-enter the aortic lumen through a secondary intimal tear.

Acute aortic dissection is a life-threatening condition with an estimated incidence of 6 per 100,000 per year. The most common risk factor is poorly controlled hypertension. Other common risk factors include a bicuspid aortic valve, pre-existing aortic disease, a family history of aortic disease, smoking, previous cardiac surgery, blunt chest trauma, and the use of psychoactive substances.

The classification of aortic dissection depends on the location of the entry tear, with the Stanford system (types A and B) and the DeBakey system (types I–III) being the most commonly used (Figure 28 in [1]).

The clinical symptoms of aortic dissection depend on the location of the tear and the involvement of the arteries branching off the aorta. The most common and characteristic symptom is sudden, severe pain in the chest, upper back, or abdomen. Stanford type A dissections, which involve the ascending aorta, have twice the mortality rate of type B dissections. The mortality rate for untreated Stanford type A dissection is approximately 50% within the first 48 h (about 1–2% per hour). Surgical intervention significantly improves survival chances, reducing the 30-day mortality rate from 90% to around 25–30%, despite the high perioperative mortality [1].

Postoperative outcomes and complications are largely influenced by the involvement of aortic branches. The most common complications include neurological deficits, mesenteric ischemia, renal failure, and lower limb ischemia, with neurological complications occurring in approximately 18% of cases [1,2].

Surgery in the acute phase of this type of aortic dissection usually involves the implantation of a vascular graft into the ascending aorta, with or without replacement of the aortic valve.

Historically, aortic dissection has been considered a contraindication for cardiac rehabilitation [3,4]. However, with the increasing number of patients undergoing surgery in the acute phase (likely due to the increased availability of CT scans in emergency departments) and the rising number of surgical survivors (due to advances in surgical and anesthetic techniques), the experience and evidence supporting rehabilitation for these patients are growing. There are few data available on the rehabilitation of patients who have residual dissection that extends more distally, beyond the section of the ascending aorta protected by a vascular prosthesis.

Objective: The objective of this study was to assess the course of rehabilitation of patients hospitalized in the cardiac rehabilitation unit after surgery for an acute Stanford type A aortic dissection, extending beyond the ascending aorta, and comparing these findings with patients who, after the same type of surgery, had no remaining dissection. Additionally, the study aimed to evaluate their one-year survival rate. The primary objective of this research was to develop a rehabilitation model for patients who survived acute Stanford type A aortic dissection, extending distally from the implanted vascular prosthesis in the ascending aorta. These patients seem to be at the highest risk.

## 2. Material and Methods

This study uses a retrospective analysis. The study group included patients who underwent surgical treatment for acute Stanford type A aortic dissection, and who had a residual aortic dissection distal to the implanted ascending aorta prosthesis. They were operated on at the Cardiac Surgery Clinic of Pomeranian Medical University (Szczecin, Poland) between June 2018 and June 2023, and were subsequently transferred directly to the cardiac rehabilitation unit.

### 2.1. Study and Comparison Groups

The study group included 37 patients who underwent surgery for an acute Stanford type A aortic dissection. Of these, 27 patients (72.97%) had a residual aortic dissection distal to the implanted ascending aorta prosthesis. The remaining 10 patients (27.03%), whose dissection was limited to the proximal ascending aorta and secured during surgery, underwent standard rehabilitation, and were included in the comparison group.

Patients excluded from the study included one patient who was discharged after five days due to an acute upper respiratory viral infection, and another patient who underwent rehabilitation without enduring trainings and was subsequently transferred to the cardiac surgery unit for further treatment. Ultimately, the study group consisted of 25 patients (15 men—60%, 10 women—40%).

The comparison group consisted of patients who underwent similar surgical procedures, including the implantation of an ascending aortic prosthesis, with or without aortic valve replacement, during the same period, and were transferred directly from the cardiac surgery unit to the cardiac rehabilitation unit. The main difference was the absence of a residual aortic dissection. Patients who additionally underwent coronary artery bypass grafting or a valve surgery other than aortic valve replacement were excluded. The comparison group included 58 patients (38 men—65.5%, 20 women—34.5%).

Characteristics of the study groups:

Comorbidities:

In the comparison group: arterial hypertension was present in 46 patients (79.3%), diabetes or impaired glucose tolerance were present in 14 patients (24.14%), lipid disorders were present in 23 patients (39.65%), chronic obstructive pulmonary disease was present in 7 patients (12%), and nicotine addiction was present in 37 patients (63.8%).

In study group: arterial hypertension was present in 23 patients (92%), diabetes or impaired glucose tolerance were present in 6 patients (2%), lipid disorders were present in 11 patients (44%), chronic obstructive pulmonary disease was present in 2 patients (8%), and nicotine addiction was present in 18 patients (72%).

The perioperative course (Table 1) and postoperative complications (Table 2) were significantly different in both groups. However, patients were compared in the Cardiac Rehabilitation Department, and our main goal was to assess whether the implementation of physical exercises and endurance training in the acute or subacute phase of aortic dissection is safe and what effects were achieved compared to the other group of patients after the same type of surgery.

### 2.2. Methods of Patient Evaluation

Upon admission to the cardiac rehabilitation unit, each patient was evaluated by a physician (both subjectively and objectively), and by a physical therapist, who assessed the patient’s functional status.

The physical therapists performed the following assessments for each patient upon admission:

- Tinetti test: this test evaluates gait and balance, with a score below 19 indicating a high risk of falling, and a score between 19 and 24 indicating a tendency to fall.
- 6-Min Walk Test (6MWT): in this test, patients walk down a hallway for six minutes and the distance traveled is measured. Patients also rate their fatigue on a 10-point Borg scale. A distance of less than 300 m indicates a significantly reduced exercise tolerance.

Patients then underwent a rehabilitation program that included:

- Respiratory exercises: active and passive exercises, and back patting (with reduced force in patients with acute aortic dissection).
- Free active exercises (anti-edema and anti-thrombotic exercises).
- General fitness exercises (individual and group sessions).
- Continuous and interval training on a cycle ergometer. The exercise started at a load of 5 watts for 5 min, with the time and load gradually increased. For patients in the study group, the load was reduced to a maximum of 50 Watts for 13 to 15 min. For other patients, the load was up to 80 Watts for 24 min, in an interval mode, with training sessions twice a day, six days a week, in morning and afternoon sessions.
- Lower limb coordination exercises using a stationary rotor.
- Stair climbing training (from the mezzanine to the second floor).

We did not perform an exercise test in the study group.

During hospitalization, patients were educated about the etiology of diseases and risk factors. Blood pressure was measured daily in the morning and evening, before and after each training session. We also corrected metabolic disorders in patients who required it. We modified the treatment of hypertension so that, in each patient, it was within the optimal range, below 130/80 mmHg.

The length of stay in the rehabilitation unit was intended to last 2–3 weeks. In the study group (patients with residual aortic dissection after surgery), two patients were returned to the cardiac surgery unit: one due to a periprosthetic hematoma around the ascending aorta, and one for further treatment.

In the comparison group, all patients were discharged home.

All patients who underwent surgery for acute aortic dissection were advised to undergo aortic CT angiography at three months (the period during which imaging stabilizes, allowing the vascular surgeon to assess the risk of adverse aortic degeneration). They were asked to do this earlier if recommended by the operator, or if clinical data warranted it. Following this, they were asked to have a consultation with the Aortic Team at our hospital. According to available data, 8 out of the 25 patients in the study group qualified for further surgical treatment.

### 2.3. Statistical Analysis

Statistical analysis was performed using Statistica 13.1 software. The analysis included determining the characteristics of the group, including medians, means, and standard deviations, as well as minimum and maximum values. A Shapiro–Wilk test was used to assess the normality of the distributions. Depending on the type of data, a Mann–Whitney U test and a Wilcoxon test were used in the statistical analysis. Results with p-values less than or equal to 0.05 were considered statistically significant.

## 3. Results

The analysis revealed that the group of patients with residual dissection was transferred to the cardiac rehabilitation unit later in the postoperative period (in the study group, the median transfer day was 10, and in the comparison group, it was 6 days). These patients achieved significantly lower initial scores in the 6MWT and Tinetti tests. However, at the final evaluation, there were no statistically significant differences between the two groups in these functional tests, although the rehabilitation in the study group was conducted with significantly lower loads (Table 3).

In the study group, 10 patients (40%) developed atrial fibrillation, and 11 patients (44%) had pericardial effusion. In the comparison group, atrial fibrillation occurred in 22 patients (37.93%), and pericardial effusion was observed in 19 patients (32.75%).

Optimal blood pressure values were obtained in 22 patients (88%) in the study group, and in 51 (87.9%) in the comparison group.

There was no statistically significant difference in one-year survival between the two groups (92% vs. 93.1% *p* = 0.961).

## 4. Discussion

Acute aortic dissection and the postoperative period are potentially dynamic states in which blood flow through the true and false lumens of the dissected aorta may change, resulting in a variable perfusion to peripheral vessels, and posing risks, such as organ perfusion disorders and limb ischemia. Optimal blood pressure control, an avoidance of strenuous physical activity, and the management of arrhythmias are critical during this period [1]. On the other hand, patients who have undergone surgery with extracorporeal circulation require physical rehabilitation. Physical activity temporarily raises systemic blood pressure, which can be unfavorable, but also improves peripheral perfusion and accelerates postoperative recovery. According to the available data and studies, a low exercise tolerance and “low cardiopulmonary capacity” are risk factors for adverse cardiovascular events in this patient group, similar to the general patient population [4,5,6,7,8,9].

Patient groups differ significantly in terms of their perioperative course. However, considering the study group to be a group of patients with a particularly high risk during rehabilitation and not having clear guidelines for this group of patients, we implemented a typical rehabilitation model with load reduction, and compared it with a group of patients after the same type of surgery because the course of rehabilitation and the observed complications often depend on the type of surgery treatment.

The current scientific literature contains numerous reports on the rehabilitation of patients after acute aortic syndromes, typically observational or retrospective studies. Often, the authors do not differentiate between patients whose aortic dissection was limited to the ascending aorta and fully secured at the time of initial surgery. Given that such patients represent a minority of those operated on for acute Stanford type A aortic dissection, it can be assumed that most studies primarily involve patients with dissections extending beyond the ascending aorta, similar to the patient group in this study [7]. A meta-analysis of five studies on the rehabilitation of patients after acute aortic syndrome evaluated a heterogeneous group of patients treated for acute aortic dissection who underwent rehabilitation according to different protocols [7]. Despite this heterogeneity, all studies demonstrated a beneficial effect of rehabilitation, primarily reflected in improved peak oxygen uptake during ergospirometry, and improved quality of life, as assessed by QoL or ADL questionnaires. However, no study was found in which the observed group was selected according to criteria identical to those used in the present study [6,7,8,10,11,12,13,14,15,16,17].

The first guidelines for the management of acute aortic syndromes were published by the Japanese Circulation Society in 2006. Subsequent updates, including those from European societies, have introduced recommendations for the rehabilitation of patients with AAD (acute aortic dissection) who have survived surgery, although standardized protocols and sufficient data on the effects of exercise on aortic dimensions and remodeling are still lacking [5]. Nevertheless, there is increasing evidence of the overall benefits of improved functional status [11,15,16,17,18,19].

Patients with acute aortic syndrome are a heterogeneous group, with varying levels of risk. Approximately 80% of these patients have hypertension, which is the primary risk factor for further adverse remodeling. There is consensus on the need for tight control of blood pressure at rest and during exercise, but no exercise targets have been established for this patient population.

Furthermore, many scientific reports focus on patients who have undergone “successful surgery”, and are referred for rehabilitation at various times, often several weeks after surgery. It appears that the highest risk group is the population observed in this study: patients with Stanford type A AAD with residual dissection, who are transferred directly from the cardiac surgery unit to the rehabilitation unit [18,19,20,21,22].

Atherosclerosis, hypertension, metabolic disorders, older age, and connective tissue diseases are the dominant causes of aortic aneurysms. Education and correction of modifiable risk factors—lipid disorders, blood pressure, diabetes, and cessation of smoking—is an essential element of rehabilitation.

This study demonstrated significant differences in the functional status of these patients upon admission to the cardiac rehabilitation unit, and substantial benefits from rehabilitation. It also showed that a typical rehabilitation program can be effectively conducted in this patient population, and results in significant functional improvement, despite the reduced exercise loads. There were no significant differences in one-year survival between the two groups.

It is worth mentioning that two patients were transferred back to the cardiac surgery unit: one was planned for further treatment due to a very narrow true lumen of the thoracic aorta, and the other was urgently transferred due to a hematoma around the ascending aorta prosthesis, which was identified during a CT scan while the patient was in the intensive care unit. Due to anemia, the scan was repeated, revealing active bleeding. It is difficult to determine what effect, if any, rehabilitation had on this complication.

It is clear that patient observations need to be extended over time and that routinely used exercise protocols should be modified. Scientific publications suggest limiting blood pressure increases during exercise to a maximum of 150/90 mmHg, with maximum loads not exceeding 4–5 MET, and lowering the target heart rate threshold. However, there are no definitive recommendations in this regard. It appears that loads in the range of 3–4 MET (expressed in Watts in this study) may be sufficient for the highest risk patients during early rehabilitation, to significantly improve functional status while being relatively safe and consistent with typical daily activities after discharge. It is undisputed that strenuous physical activities, especially static ones, are inadvisable for this group of patients [7,8,9,22].

This study is limited by the small sample size of patients and the single-center design. Additionally, the observation period may need to be significantly extended to draw more definitive conclusions.

It is also worth mentioning the lack of any significant differences in the functional status at discharge from the Cardiac Rehabilitation Department in both groups, despite there being significant differences in the perioperative course. The differences would probably be visible in a stress test (electrocardiographic stress test or ergospirometry); however, we did not perform either of these tests for the study group.

## 5. Conclusions

Our study indicates that patients who have undergone surgery for acute aortic dissection, still have remaining dissection, and who are transferred directly from the cardiac surgery unit to the cardiac rehabilitation unit can be improved using a similar protocol to other patients who have undergone similar surgery. However, the training loads and achievable goals need to be adjusted. Nevertheless, further multicenter studies, utilizing a follow-up covering a long period, are necessary to develop an optimal rehabilitation protocol for this patient population.

## Figures and Tables

**Table 1:** Perioperative data.

	Comparison Group n = 58			Study Group n = 25			*p*
	Me	Q1	Q3	Me	Q1	Q3	
Perfusion time [min]	96.00	78.00	129.00	144.00	109.00	182.00	<0.001
Clamping time [min]	68.50	58.00	95.00	75.00	65.00	102.00	0.098
Intubation time [min]	555.00	350.00	735.00	840.00	555.00	1440.00	0.001
Number of transfused RBCs [units]	2.00	0.00	2.00	4.00	2.00	5.00	0.000
FFP quantity [units]	2.00	0.00	3.00	3.00	2.00	4.00	0.053
Number of plate units [milliliters]	0.00	0.00	250.00	250.00	0.00	300.00	0.001
Drainage [milliliters]	430.00	320.00	630.00	800.00	500.00	1300.00	0.003

Me—median; Q1—first quartile; Q3—third quartile; *p*—statistical significance, n—sample size.

**Table 2:** Complications in the postoperative period.

	Comparison Group n = 58	Study Group n = 25
Renal failure requiring hemofiltration or dialysis	2 (3.44%)	6 (24%)
Respiratory failure	3 (5.17%)	6 (24%)
Pneumonia	11 (18.96%)	7 (28%)
Delirium	8 (13.79%)	6 (24%)
Stroke	3 (5.17%)	3 (12%)

**Table 3:** Comparison of the two patient groups. Group A—comparison group (58 patients), Group B—study group (25 patients).

Variables	Group A, n = 58			Group B, n = 25			*p*
	Me	Q1	Q3	Me	Q1	Q3	
Age [years]	64.76	55.37	70.18	59.90	51.38	69.17	0.264
Body height [cm]	171.50	164.50	176.00	173.00	164.00	178.00	0.560
Body weight [kg]	83.50	74.75	94.75	80.00	6900	90.00	0.163
BMI [kg/m^2^]	29.61	25.93	32.50	26.45	23.81	29.74	0.034
6MWT admission [m]	341.00	192.50	396.00	220.00	110.00	305.50	0.019
6MWT discharge [m]	451.00	363.00	528.00	423.50	300.00	464.75	0.096
Tinetti test admission	26.00	25.00	27.00	24.00	22.00	25.00	<0.001
Tinetti test discharge	28.00	27.00	28.00	28.00	25.75	28.00	0.061
Watt	60.00	50.00	80.00	40.00	37.50	40.00	<0.001

Me—median; Q1—first quartile; Q3—third quartile, *p*—statistical significance, n—sample size, Watt—maximum workload.

## Data Availability

The raw data supporting the conclusions of this article will be made available by the authors on request.

## References

[1] ESC Scientific Document Group (2024). 2024 ESC Guidelines for the management of peripheral arterial and aortic diseases. Eur. Heart J..

[2] Cornelis J., Myers J., Heidbuchel H., Vrints C., Beckers P. (2018). Exercise Training in Heart Failure Patients With Persistent Atrial Fibrillation: A Practical Approach. Card. Fail. Rev..

[3] Alegria-Barrero E., Alegria-Ezquerra E. Cardiac Rehabilitation: Evidence for Action. https://www.escardio.org/Journals/E-Journal-of-Cardiology-Practice/Volume-11/Cardiac-rehabilitation-Evidence-for-Action.

[4] Czerny M., Grabenwöger M., Berger T., Aboyans V., Della Corte A., Chen E.P., Desai N.D., Dumfarth J., Elefteriades J.A., Etz C.D. (2024). EACTS/STS Scientific Document Group. EACTS/STS Guidelines for diagnosing and treating acute and chronic syndromes of the aortic organ. Eur. J. Cardio-Thorac. Surg..

[5] Fuglsang S., Heiberg J., Hjortdal V.E., Laustsen S. (2017). Exercise-based cardiac rehabilitation in surgically treated type-A aortic dissection patients. Scand. Cardiovasc. J..

[6] Pasadyn S.R., Roselli E.E., Artis A.S., Pasadyn C.L., Phelan D., Blackstone E.H. (2021). From Court to Couch: Exercise and Quality of Life after Acute Type A Aortic Dissection. Aorta.

[7] Carbone A., Lamberti N., Manfredini R., Trimarchi S., Palladino R., Savriè C., Marra A.M., Ranieri B., Crisci G., Izzo R. (2024). Cardiac rehabilitation and acute aortic dissection: Understanding and addressing the evidence GAP a systematic review. Curr. Probl. Cardiol..

[8] Zhou N., Mampuya W.M., Iliou M.C. (2022). Is Exercise Blood Pressure Putting the Brake on Exer-cise Rehabilitation after Acute Type A Aortic Dissection Surgery?. J. Clin. Med..

[9] Zhou N., Fortin G., Balice M., Kovalska O., Cristofini P., Ledru F., Mampuya W.M., Iliou M.C. (2022). Evolution of Early Postoperative Cardiac Rehabilitation in Patients with Acute Type A Aortic Dissection. J. Clin. Med..

[10] Lin Y., Liang T., Zhang X., Peng Y., Li S., Huang X., Chen L. (2023). Early goal-directed mobilization in patients with acute type A aortic dissection: A randomized controlled trial. Clin. Rehabil..

[11] Nakamura K., Ohbe H., Uda K., Matsui H., Yasunaga H. (2022). Effectiveness of early rehabilitation following aortic surgery: A nationwide inpatient database study. Gen. Thorac. Cardiovasc. Surg..

[12] Chaddha A., Kline-Rogers E., Braverman A.C., Erickson S.R., Jackson E.A., Franklin B.A., Woznicki E.M., Jabara J.T., Montgomery D.G., Eagle K.A. (2015). Survivors of Aortic Dissection: Activity, Mental Health, and Sexual Function. Clin. Cardiol..

[13] Hirakawa K., Nakayama A., Saitoh M., Hori K., Shimokawa T., Iwakura T., Haraguchi G., Isobe M. (2022). Related to Hospitalisation-Associated Disability in Patients after Surgery for Acute Type A Aortic Dissection: A Retrospective Study. Int. J. Environ. Res. Public Health.

[14] Tashima Y., Toyoshima Y., Chiba K., Nakamura N., Adachi K., Inoue Y., Yamaguchi A. (2021). Physical activities and surgical outcomes in elderly patients with acute type A aortic dissection. J. Card. Surg..

[15] Corone S., Iliou M.C., Pierre B., Feige J.M., Odjinkem D., Farrokhi T., Bechraoui F., Hardy S., Meurin P., Cardiac Rehabilitation Working Group of the French Society of Cardiology (2009). French registry of cases of type I acute aortic dissection admitted to a cardiac rehabilitation center after surgery. Eur. J. Cardiovasc. Prev. Rehabil..

[16] Feng D., Ke J., Huang S., Lang X. (2021). A scoping review of exercise-based cardiac rehabilitation for patients with aortic dissection. Rev. Cardiovasc. Med..

[17] Jepsen L.R., D’Oria M., Pedersen S.F., Budtz-Lilly J. (2023). Efficacy and Safety of Exercise Testing and Rehabilitation for Aortic Dissection Patients: A SCOPING REVIEW. J. Cardiopulm. Rehabil. Prev..

[18] Van Iterson E.H., Laffin L.J., Svensson L.G., Cho L. (2022). Individualized exercise prescription and cardiac rehabilitation following a spontaneous coronary artery dissection or aortic dissection. Eur. Heart J. Open.

[19] Spanos K., Tsilimparis N., Kölbel T. (2018). Exercise after Aortic Dissection: To Run or Not to Run. Eur. J. Vasc. Endovasc. Surg..

[20] Hornsby W.E., Norton E.L., Fink S., Saberi S., Wu X., McGowan C.L., Brook R.D., Jones L.W., Willer C.J., Patel H.J. (2020). Cardiopulmonary Exercise Testing Following Open Repair for a Proximal Thoracic Aortic Aneurysm or Dissection. J. Cardiopulm. Rehabil. Prev..

[21] Schwaab B., Rauch B., Völler H., Benzer W., Schmid J.P. (2022). Beyond randomised studies: Recommendations for cardiac rehabilitation following repair of thoracic aortic aneurysm or dissection. Eur. J. Prev. Cardiol..

[22] Carbone A., Palladino R., Franzese M., Castaldo R., Ranieri B., Crisci G., Izzo R., Esposito G., Cittadini A., Schreurs B. (2024). Health-related quality of life in patients with aortic dissection: An unmet need. Curr. Probl. Cardiol..

